# Supplementary material for: Splice-Junction-Based Mapping of Alternative Isoforms in the Human Proteome
Source: Cell Rep. Author manuscript; Available in PMC 2020 Jan 15. (PMC6961840; doi:10.1016/j.celrep.2019.11.026)
Supplement: 2 [file NIHMS1546469-supplement-2.zip › DF1/PXD010154/Liver/Liver_42_MINK1_AATAAGAGAAEAAGEGAAAAAGGHAGSAAGGGAAAG.pdf]

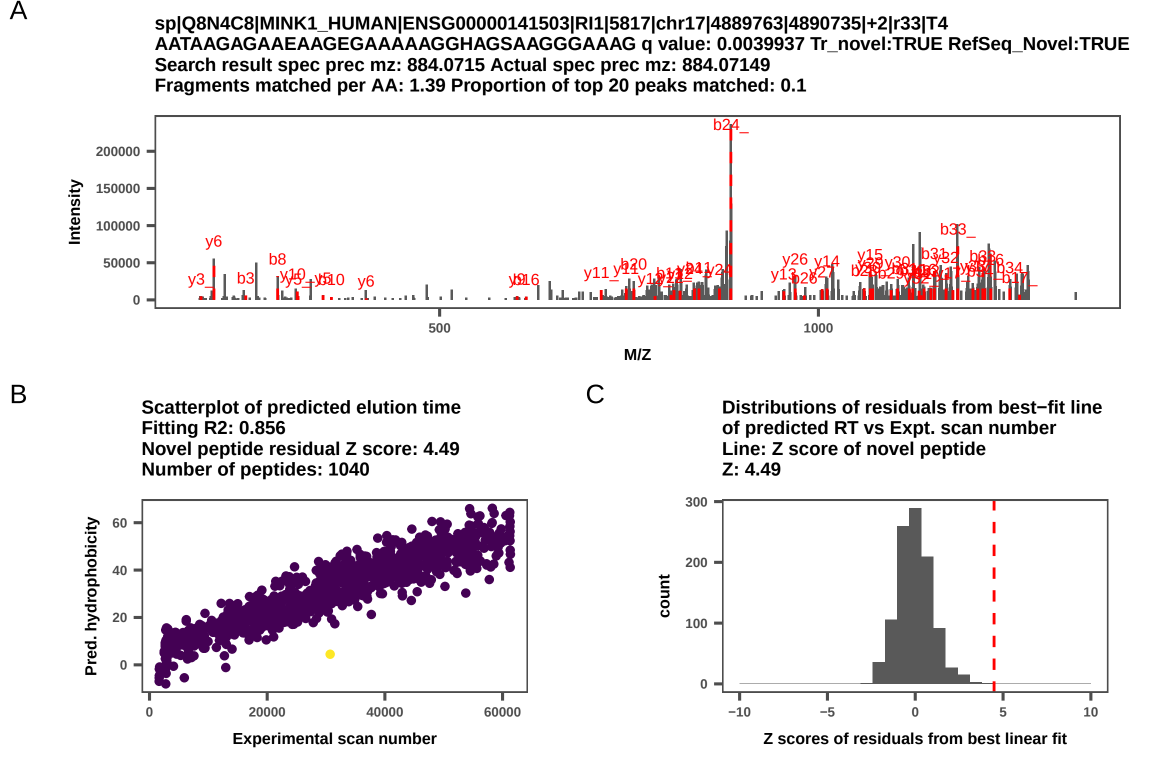
A
sp|Q8N4C8|MINK1_HUMAN|ENSG00000141503|RI1|5817|chr17|4889763|4890735|+2|r33|T4
AATAAGAGAAEAAGEGAAAAAGGHAGSAAGGGAAAG q value: 0.0039937 Tr_novel:TRUE RefSeq_Novel:TRUE
Search result spec prec mz: 884.0715 Actual spec prec mz: 884.07149
Fragments matched per AA: 1.39 Proportion of top 20 peaks matched: 0.1
Intensity
200000
150000
100000
50000
0
b24_
b33_
y6
y3_
b3
b8
y10
y6
y11_
b20
y26
y14
y13
y15
b31
M/Z
500
1000
B
Scatterplot of predicted elution time
Fitting R2: 0.856
Novel peptide residual Z score: 4.49
Number of peptides: 1040
Pred. hydrophobicity
60
40
20
0
Experimental scan number
0
20000
40000
60000
C
Distributions of residuals from best−fit line
of predicted RT vs Expt. scan number
Line: Z score of novel peptide
Z: 4.49
count
300
200
100
0
Z scores of residuals from best linear fit
−10
−5
0
5
10
